# Automated tumor proportion scoring for PD-L1 expression based on multistage ensemble strategy in non-small cell lung cancer

**DOI:** 10.1186/s12967-021-02898-z

**Published:** 2021-06-07

**Authors:** Boju Pan, Yuxin Kang, Yan Jin, Lin Yang, Yushuang Zheng, Lei Cui, Jian Sun, Jun Feng, Yuan Li, Lingchuan Guo, Zhiyong Liang

**Affiliations:** 1 Department of Pathology, Molecular Pathology Research Center, Peking Union Medical College Hospital, Chinese Academy of Medical Sciences and Peking Union Medical College, Beijing, 100730 China; 2 School of Information Science and Technology, Northwest University, Xi’an, Shanxi China; 3 Department of Pathology, Fudan University Shanghai Cancer Center, Shanghai, China; 4 School of Engineering, Westlake University, Hangzhou, Zhejiang China; 5 Department of Pathology, the First Affiliated Hospital of Soochow University, Suzhou, Jiangsu China

**Keywords:** PD-L1, NSCLC, Automated scoring, TPS, Multistage ensemble strategy

## Abstract

**Introduction:**

Programmed cell death ligand-1 (PD-L1) expression is a promising biomarker for identifying treatment related to non-small cell lung cancer (NSCLC). Automated image analysis served as an aided PD-L1 scoring tool for pathologists to reduce inter- and intrareader variability. We developed a novel automated tumor proportion scoring (TPS) algorithm, and evaluated the concordance of this image analysis algorithm with pathologist scores.

**Methods:**

We included 230 NSCLC samples prepared and stained using the PD-L1(SP263) and PD-L1(22C3) antibodies separately. The scoring algorithm was based on regional segmentation and cellular detection. We used 30 PD-L1(SP263) slides for algorithm training and validation.

**Results:**

Overall, 192 SP263 samples and 117 22C3 samples were amenable to image analysis scoring. Automated image analysis and pathologist scores were highly concordant [intraclass correlation coefficient (ICC) = 0.873 and 0.737]. Concordances at moderate and high cutoff values were better than at low cutoff values significantly. For SP263 and 22C3, the concordances in squamous cell carcinomas were better than adenocarcinomas (SP263 ICC = 0.884 vs 0.783; 22C3 ICC = 0.782 vs 0.500). In addition, our automated immune cell proportion scoring (IPS) scores achieved high positive correlation with the pathologists TPS scores.

**Conclusions:**

The novel automated image analysis scoring algorithm permitted quantitative comparison with existing PD-L1 diagnostic assays and demonstrated effectiveness by combining cellular and regional information for image algorithm training. Meanwhile, the fact that concordances vary in different subtypes of NSCLC samples, which should be considered in algorithm development.

**Supplementary Information:**

The online version contains supplementary material available at 10.1186/s12967-021-02898-z.

## Introduction

Programmed cell death-1 (PD-1) and programmed cell death ligand-1 (PD-L1) pathway have emerged as immune checkpoints in several malignancies including lung carcinoma [1]. PD-L1 is expressed in many tumors cells (TCs), which can interact with PD-1 expressed on cytotoxic T cells and thereby evade the recognition by the host’s immune system [2, 3]. PD-1/PD-L1 inhibitors are used to block the immuno-escape interaction between TCs and immune cells in a variety of cancers. [4] Assessment of PD-L1 expression and prediction the treatment outcomes of PD-1/PD-L1 inhibitors is a critical part of patient management in [5–8]. However, different antibody clones and diagnosis platforms exist for the immunohistochemistry (IHC) assays in PD-L1 diagnosis [9–12]. The Food and Drug Administration (FDA) and European Medicines Agency (EMA) have approved several diagnostic IHC antibodies with respective platforms, such as 22C3, 28-8, 73-10 from Dako (Agilent), SP142, SP263 from Ventana Medical Systems, to assess PD-L1 expression levels in patients with non-small cell lung cancer (NSCLC) [9, 13, 14].

However, each IHC assay has different scoring methods and cutoff values to predict the PD-L1 status of a tumor. In the SP263 assay, tumor proportion score (TPS) ≥ 25% is used as the cutoff, and in the 28-8 and 22C3 assays, TPS ≥ 1% is used as the cutoff to predict PD-L1 positivity in NSCLC [9, 15]. A few studies have evaluated various IHC assays for their reproducibility and sensitivity based on respective scoring criteria and cutoff values of PD-L1 assays [13, 16]. Strong concordance was found at various cutoff values with 22C3, 28-8, and SP263 assays, lower sensitivity was reported in the SP142 assay [13]. In addition, manual PD-L1 scoring by different pathologists might lead to inconsistent results. Previous studies demonstrated inter-pathologist variability could be even higher than assay variability due to the subjective nature of IHC reporting [9, 17, 18]. Accurate PD-L1 scoring was even more difficult in tissue samples with low expression (< 10%) and in assays with 1%, 25%, or 50% cutoff value [17, 19], and further obstacles include weak-staining TCs, PD-L1–positive immune cells (ICs; lymphocytes and macrophages), and cytoplasm-staining TCs in PD-L1 scoring [20, 21]. These staining result in false positive signals and unfaithful PD-L1 scoring which cannot be rectified by experienced pathologists. In summary, IHC-based PD-L1 scoring is hindered by tedious, subjective, and time consuming process of manual scoring and the inconsistence of results amony pathologists [15, 21].

Compared with manual scoring by pathologists, automated image analysis may provide an aided scoring tool for pathologists to reduce inter- and intrareader variability and increase scoring throughput (e.g., high efficiency by eliminating the need for manual area selection on stained samples) [15, 21, 22]. Recently, many researchers have demonstrated the feasibility of deep learning-based methods in estimating TPS automatically [15, 21], these algorithms could be categorized into regional area ratio-based and cellular count ratio-based methods. The regional area ratio-based method estimates TPS by calculating the ratio between region areas of positive TCs [TC (+)] and TCs [15, 21], which was not well suited with the current clinical guidelines. TPS was recommended to be calculated on the basis of tumor cellular count [23]. On the other hand, cellular count ratio-based methods, which directly extracted cellular information at high magnification scale to localize and count the cells, however, the accuracy of the algorithm needs further improvement [24].

In clinical diagnosis, pathologists approximately distinguish the TC region from other regions firstly at the lower magnification scale and then zoom into the higher magnification for accurate cell counting. Such a process works best to prevent both false positives and false negatives (e.g., histocytes and necrotic cells) by obtaining both the regional-and-cellular information. Motivated by this clinical diagnosis process in practice, we developed an automated tumor proportion scoring method using a multi-stage ensemble strategy. Taking advantage of both methods mentioned above, we designed a framework composed of a cellular localization network (C-Net) and a regional segmentation network (R-Net), and the efficacy of this algorithm was compared with PD-L1 scoring performed by experienced pathologists.

## Materials and methods

### Tumor samples and assays

Archived, commercially sourced, formalin-fixed paraffin-embedded NSCLC sections (N = 230) were obtained from the pathology departments of three hospitals, namely Peking Union Medical College Hospital, Fudan University Shanghai Cancer Center, and the First Affiliated Hospital of Soochow University, China. The samples were prepared and stained using the Ventana PD-L1 (SP263) assay (Cat#07494190001, Ventana Medical Systems, Inc., Tucson, USA) using the automated Ventana BenchMark Ultra platform, according to the manufacturer’s protocol. At the same time, among the 230 sections, 117 samples, from Peking Union Medical College Hospital, were stained using the Dako PD-L1 (22C3) pharmDx assay (Cat#SK006, Heverlee, Belgium) using the Dako Autostainer Link48 platform. The PD-L1–stained TCs were scored with TPS, which represents the best estimated percentage (0–100%) of TCs showing partial or complete membranous PD-L1 staining. At least 2 pathologists trained on the use of Ventana PD-L1 (SP263) assay and Dako PD-L1 (22C3) assay scored the proportion of tumor cells (TCs) with PD-L1 membrane staining to obtain a consistent TPS value.

### Image analysis scoring algorithm

An image analysis scoring algorithm was proposed, which consisted of 2 main parts: (1) cellular localization and elementary TPS calculation using fully convolutional networks, using a weighted pixel-wise cross-entropy, (2) a synchronized regional segmentation branch to refine the TPS.

### Cellular localization algorithm

Cellular localization aimed to utilize fully convolutional networks to quantitatively classify, localize, and count the PD-L1 cell nuclei. However, the excessive decoding processes of excited algorithms (e.g., FCN [24] and U-Net [25]) significantly increased the parameters. Thus, to obtain results in a timely manner, we designed the C-Net with a high-efficiency decoder that restored the resolution of the encoded features. C-Net utilizes the deep supervision method and transition blocks to allow the kernels in lower convolutional layers to extract higher level semantic features, which were critical for prediction [26]. Moreover, we proposed a weighted pixel-wise cross-entropy as a loss function of C-Net to promote the algorithm in the right direction.

## Weighted pixel-wise cross-entropy loss

Tumor cells were close and/or adhesive, which made the network recognizing multiple cells as one cell easily. Meanwhile, the cross-entropy was ineffective since the point-level annotation could not represent cells with rupturing membranes or missing nucleus. For identifying the tumor cells individually, we constructed a weight matrix $\varphi$ which increased the loss of those difficult cells during training effectively. It could also be understood as a kind of difficult sample mining. The weight $\varphi$ was defined as:

1 $$ \varphi \left( {Y_{b}^{i} ,\hat{Y}_{b}^{i} } \right) = \lambda ||Y_{b}^{i}  - \hat{Y}_{b}^{i} ||^{\gamma }  $$

where $\hat{Y}_{b}^{i}$ denoted the ground truths of the pixel *i* in flattened *b*th image and $Y_{b}^{i}$ was the predicted probability. Lin, Tsung-Yi et al utilized tunable focusing parameters to balance the importance of positive/negative examples in focal loss [27]. Hence, we also utilized two tunable focusing parameter *λ* and *γ* to weight the importance of matrix $\varphi$ for the weighted pixel-wise cross-entropy loss *L*, respectively. In our experiments we set *λ* = 3 and *γ* = 3. Specifically, $\varphi$ made the false prediction pixels with a higher loss. Accordingly, the *L* could be formulated as:

2 $$ \begin{gathered}   E = \varphi (Y_{b}^{i} ,\hat{Y}_{b}^{i} )[Y_{b}^{i} \log \hat{Y}_{b}^{i}  + (1 - Y_{b}^{i} )\log (1 - \hat{Y}_{b}^{i} )], \hfill \\   L =  - \frac{1}{B}\frac{1}{N}\sum\limits_{{b = 1}}^{B} {\sum\limits_{{i = 1}}^{N} E }  \hfill \\  \end{gathered}  $$

where B indicated the batch size, and N indicated the number of pixels of each image. Further, each $Y_{b}^{i}$ was obtained by using 1 × 1 convolutions with sigmoid activation. In this sense, the C-Net down-weighted easy examples with lower loss and focused on training hard examples with higher loss. It induced that the training of C-Net would be stabilized in the right direction.

## Regional segmentation and TPS refinement

Furthermore, we employed DeeplabV3+ pre-trained on ImageNet as the basic model for the regional segmentation network (R-Net) to generate a tumor region probability map on a low magnification scale. The map was used to weigh out the features in the C-Net. Owing to this, the nontumor cell features were suppressed and the cell got a minimal probability value after the activation layer.

Other comparable cellular localization algorithms were obtained from the previous studies, including Mi [24], U-Net [25], and *S*^3^Net [26]. A complete image analysis was composed of the algorithm and its training data set. Because of the lack of original training data sets in the previous studies, we could not reproduce previous image analyses completely. Therefore, we re-trained the three previous algorithms using our cell data sets and compared the effectiveness of cellular localization of these four algorithms.

At the same time, we combined our R-Net with four cellular localization algorithms respectively and scored TPS values on big patches (size 4096 × 4096), sampled from whole slide images (WSIs), for evaluating the effectiveness of our R-Net. The pathologist scores, annotated as the ground truth data, of big patches were scored by two manufacturer-trained pathologists, and a consistent value was obtained. Slides were scanned on a NanoZoomer 2.0HT scanner at 40× magnification.

### Statistical analysis

The results of cellular localization algorithms were evaluated using 4 indexes [25]: the accuracy of the detection of TC (+) and negative tumor cells [TC (−)] (Object F1 Score), the accuracy of the count of TC (+) and TC (−) [the mean absolute error (MAE), the root mean squared error (RMSE) and the mean absolute percent error (MAPE)]. These four indexes are generally utilized to evaluate the cellular localization algorithms. Moreover, the effectiveness of R-Net was evaluated using six indexes: MAE, RMSE, MAPE, the Pearson product-moment correlation coefficient, Spearman’s rank correlation coefficient, and intraclass correlation coefficient (ICC).

To assess the similarity in TPS values between image analysis and pathologist, Fleiss’ kappa statistics for categorical scores were used after dichotomization based on specified cutoffs. The various cutoff values utilized were 1%, 5%, 10%, 25%, and 50%. All of these values have been previously used in various immune checkpoint inhibitor trials or as suggested by the manufactures [13]. The ICC analysis was used to assess scoring reliability for continuous TPS values. ICCs of 0.75 to 0.9 and > 0.9 were considered to indicate good and excellent reliability, respectively [28]. Kappa scores of ≥ 0.8 were considered near perfect, scores of 0.60–0.79 were considered strong, scores of 0.40–0.59 were considered moderate, and scores of 0.20–0.39 were considered weak. SPSS software, version 25.0 (IBM Corporation), was used for statistical analyses, where *P* < 0.05 was considered statistically significant.

### Optimization of image analysis scoring algorithm

**Fig. 1:**
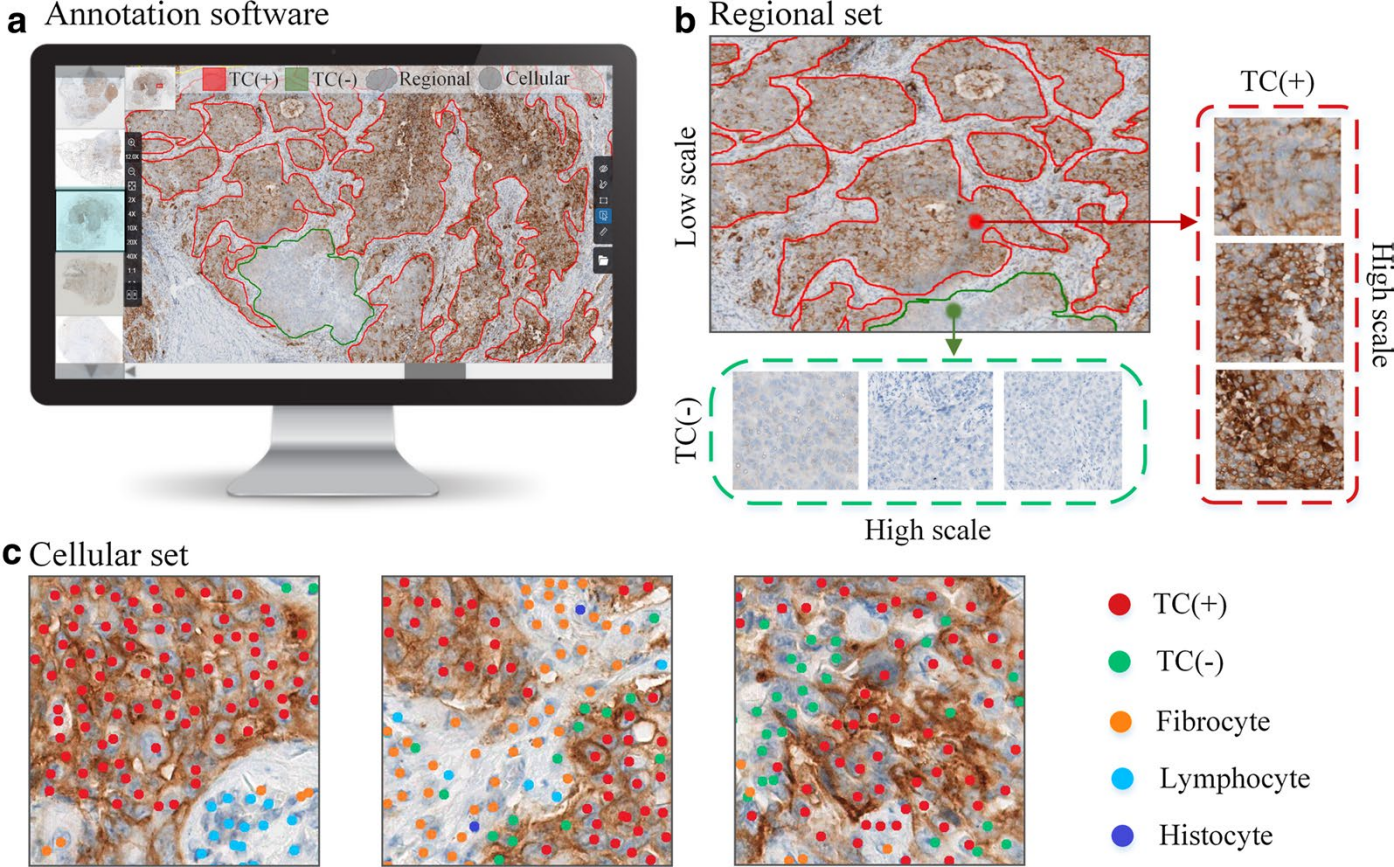
Procedure for annotation. **a** A computer-aided program was designed for pathologist’s annotation; **b** Pathologists annotated regional information for the regional set, including TC (+) regions, TC (−) regions, and normal regions; **c** Cellular annotation included TC (+) (red), TC (−) (green), fibrocyte (orange), lymphocyte (blue), and histocyte (purple). *TC* tumor cells

A computer-aided program was developed for manual annotation for pathologists (Fig. 1) where they utilized 30 PD-L1(SP263)-staining cases, including 15 squamous cell carcinoma cases and 15 adenocarcinoma cases, to sample patches for annotation from WSIs. Cell tags were labeled on 4264 patches of size 512 × 512 pixels, which represented the type of TC in the 40× magnification scale and consisted of 519275 TC (+), 537471 TC (−), and 693290 normal cells (e.g., histocyte, lymphocyte, and fibrocyte). Region tags were labeled on 596 patches of size 2048 × 2048 pixels, which represented the type of tumor region on the 10× magnification scale and consisted of 272 TC (+) regions, 584 TC (−) regions, and 486 normal regions. The patches of cell tags and region tags were extracted nonrepetitively. Approximately 60% of cell and regions tags were designated as the training data set, 20% as validating data set, and 20% as testing data set.

**Fig. 2:**
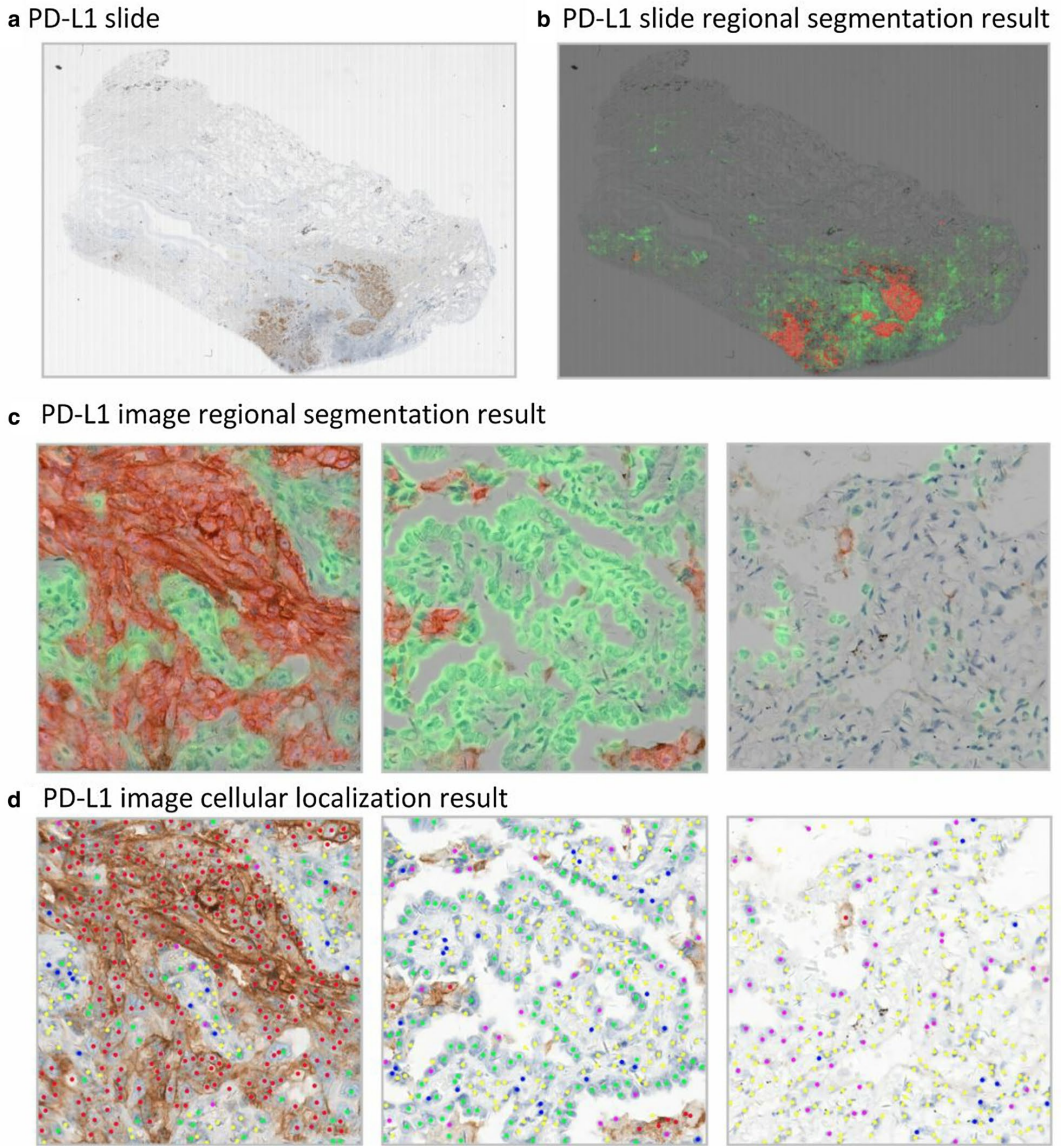
Image analysis result of a case. **a** PD-L1 slide representing original image; **b** PD-L1 slide representing regional segmentation of the whole slide; **c** PD-L1 image representing regional segmentation result; **d** PD-L1 image representing cellular localization results. In regional segmentation, red and green areas represent TC (+) and TC (−) regions separately. In cellular localization, TC (+) (red), TC (−) (green), fibrocyte (yellow), lymphocyte (blue), and histocyte (pink) are shown separately. *PD-L1* programmed death-ligand 1, *TC* tumor cells

To test the robustness of this approach and avoid over-fitting of deep neural networks, online data augmentation techniques, including random rotation, shear, shift, zooming of width and height, whitening, and horizontal and vertical flips, were employed to enlarge the training set. Both C-Net and R-Net were optimized by the momentum optimizer with a batch size of 4, an initial learning rate of 0.001, and maximum epoch of 200. Eventually, the image analysis achieved regional segmentation and cellular localization on WSIs and automated TPS of the whole slides. The result obtained after image analysis optimization for a case is presented in Fig. 2.

### Optimization for immune cells PD-L1 evaluation

Except for PD-L1 evaluation of tumor cells, we also optimized our algorithm for immune cells PD-L1(SP263) evaluation. The immune cells included lymphocytes and histocytes in tumor regions. The PD-L1–stained ICs were scored with immune cell proportion scoring (IPS), which represents the estimated percentage (0–100%) of ICs showing membranous or cytoplasmic PD-L1 staining. To assess the correlation between automated IPS and TPS, we used Mann–Whitney *U* test, chi-square test, Spearman and Pearson correlation coefficients for evaluation.

## Results

### Tumor samples demographics

Tumor resection samples for PD-L1 assessment were obtained from 230 patients with stage I to IV NSCLC.

30 cases were used for algorithm training. After exclusion of slides with poor staining and scanning quality, there were eventually 192 NSCLC PD-L1(SP263) staining slides for image analysis, including 88 squamous cell carcinoma cases, 100 adenocarcinoma cases, and four adenosquamous carcinoma cases. According to the manual scoring results, there were 67 PD-L1–negative cases (TPS < 1%; 18 squamous cell carcinomas cases, 48 adenocarcinomas cases and 1 adenosquamous carcinomas cases), 52 low expression cases (1% ≤ TPS < 25%; 24 squamous cell carcinomas cases and 28 adenocarcinomas cases), 13 moderate expression cases (25% ≤ TPS < 50%; four squamous cell carcinomas cases and nine adenocarcinomas cases), and 60 high expression cases (TPS ≥ 50%; 41 squamous cell carcinomas cases, 16 adenocarcinomas cases, and three adenosquamous carcinomas cases).

Meanwhile, 117 NSCLC PD-L1(22C3) staining slides included 47 squamous cell carcinoma cases, 66 adenocarcinoma cases, and four adenosquamous carcinoma cases. According to the manual scoring results, there were 65 PD-L1–negative cases (TPS < 1%; 18 squamous cell carcinomas cases, 46 adenocarcinomas cases and one adenosquamous carcinomas cases), 11 low expression cases (1% ≤ TPS < 25%; seven squamous cell carcinomas cases and four adenocarcinomas cases), nine moderate expression cases (25% ≤ TPS < 50%; one squamous cell carcinomas cases and eight adenocarcinomas cases), and 32 high expression cases (TPS ≥ 50%; 21 squamous cell carcinomas cases, eight adenocarcinomas cases, and three adenosquamous carcinomas cases).

### Comparison between pathologist scores and image analysis scores

**Fig. 3:**
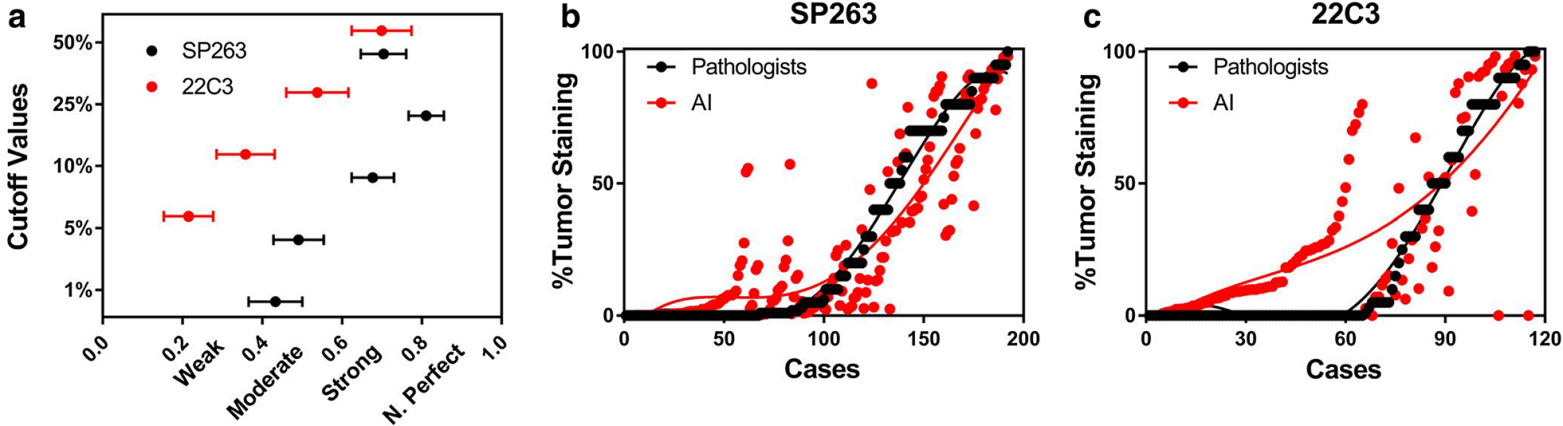
**a** Concordance of scoring tumor cell PD-L1 expression using Fleiss’ kappa statistics at 1%, 5%, 10%, 25%, and 50% cutoffs; Comparison between image analysis and pathologist scores in TPS values in SP263 (**b**) and 22C3 (**c**). *PD-L1* programmed death-ligand 1, *TPS* tumor proportion score, *AI* artificial intelligence

In PD-L1(SP263) staining slides, the automated image analysis achieved high concordance with pathologist scores (ICC: 0.873, 95% CI 0.835–0.903; Table 1). The PD-L1 expression on the basis of TC scores across all the cutoffs analyzed revealed strong comparable concordances at 10% (κ: 0.677, 95% CI 0.624–0.730; Fig. 3a), 25% (κ: 0.811, 95% CI 0.767–0.855; Fig. 3a), and 50% (κ: 0.704, 95% CI 0.647–0.761; Fig. 3a) cutoffs. However, at 1% and 5% cutoff values, the concordances were relatively low (1% κ: 0.433, 95% CI 0.366–0.500; 5% κ: 0.491, 95% CI 0.428–0.554; Fig. 3a).

**Table 1:** The concordance (intraclass correlation coefficient) of multistage net in different histological variants

		ICC	95% CI	*P*
SP263	Total	0.873	0.835–0.903	0.000
	SCC	0.884	0.828–0.923	0.000
	Ad	0.795	0.710–0.856	0.000
22C3	Total	0.737	0.641–0.810	0.000
	SCC	0.782	0.641–0.873	0.000
	Ad	0.500	0.295–0.661	0.000

*CI* confidence interval, *ICC* intraclass correlation coefficient, *SCC* squamous cell carcinomas, *Ad* adenocarcinomas

The concordance values between pathologist scores and image analysis scores were also satisfactory (ICC: 0.737, 95% CI 0.641–0.810; Table 1) in PD-L1 (22C3) staining slides. In various cutoffs evaluations, the results revealed moderate and strong concordances at 25% (κ: 0.538, 95% CI 0.460–0.616; Fig. 3a) and 50% (κ: 0.699, 95% CI 0.624–0.774; Fig. 3a). However, the concordances at 5% and 10% cutoff values were weak (5% κ: 0.215, 95% CI 0.153–0.277; 10% κ: 0.358, 95% CI 0.285–0.431; Fig. 3a). At 1% cutoff value, there was no concordance between pathologist scores and image analysis scores.

In both SP263 and 22C3 staining, we noticed that in PD-L1–negative expression cases, the image analysis scores were higher than pathologist scores. However, the pathologist scores were much higher in cases with moderate and high PD-L1 expression (Fig. 3b, c). Meanwhile, we noticed that when comparing with PD-L1(SP263) staining results, the differences between pathologists and image analysis were more drastic in PD-L1(22C3) negative expression cases (Fig. 3c).

### Comparison between pathologist scores and image analysis scores in different histological subtypes

**Fig. 4:**
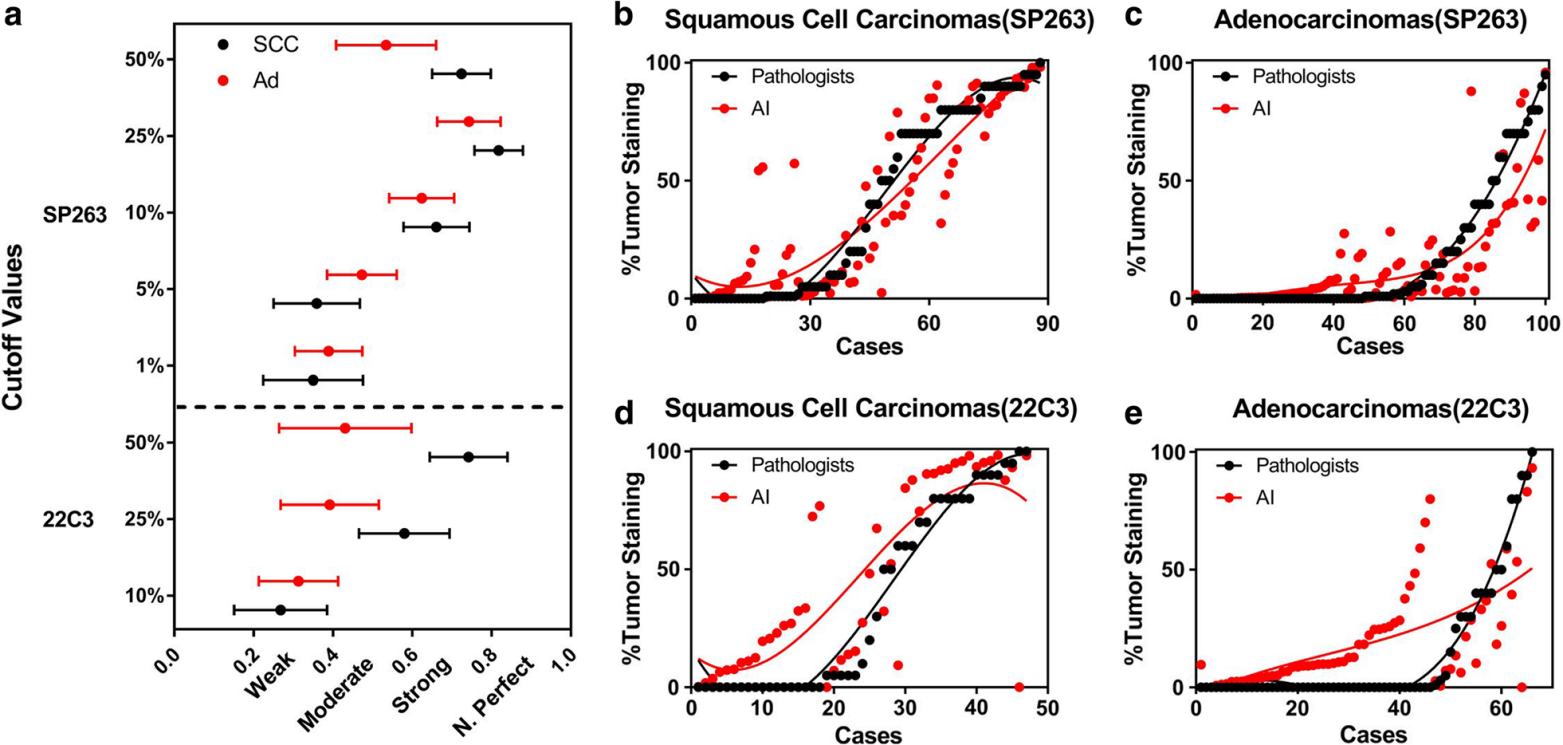
**a** Concordance of scoring tumor cell PD-L1 expression in SCC and adenocarcinomas using Fleiss’ kappa statistics at 1%, 5%, 10%, 25%, and 50% cutoffs; Comparison between image analysis and pathologist scores in PD-L1(SP263) SCC cases (**b**) and adenocarcinoma cases (**c**); Comparison between image analysis and pathologist scores in PD-L1(22C3) SCC cases (**d**) and adenocarcinoma cases (**e**). *PD-L1* programmed death-ligand 1, *SCC* squamous cell carcinomas, *Ad* adenocarcinomas, *AI* artificial intelligence

In SP263 staining slides, the correlation of concordances values between pathologist scores and image analysis scores was evaluated in 88 squamous cell carcinoma cases and 100 adenocarcinoma cases, respectively. On the basis of the TPS values, concordances in squamous cell carcinomas were better than adenocarcinomas (ICC = 0.884 vs 0.783; Table 1). However, evaluation at different cutoffs revealed that the concordances in adenocarcinomas were better than those in squamous cell carcinomas at low cutoff values (1% κ: 0.350 vs 0.389; 5% κ: 0.359 vs 0.473; Fig. 4a). Nevertheless, the concordances in squamous cell carcinomas were high when compared at moderate and high cutoff values (10% κ: 0.661 vs 0.624; 25% κ: 0.818 vs 0.743; 50% κ: 0.724 vs 0.534; Fig. 4a).

Similar to SP263, in 47 squamous cell carcinoma cases and 66 adenocarcinoma PD-L1(22C3) staining cases, concordances in squamous cell carcinomas were better than adenocarcinomas (ICC = 0.782 vs 0.500; Table 1). Moreover, the concordances in squamous cell carcinomas were highly satisfactory when compared at moderate and high cutoff values (25% κ: 0.580 vs 0.392; 50% κ: 0.742 vs 0.431; Fig. 4a). At 10% cutoff value, the concordances in adenocarcinomas were better (10% κ: 0.268 vs 0.313; Fig. 4a). Nevertheless, at 1% and 5% cutoff values, there were no concordances between pathologist scores and image analysis scores in either adenocarcinomas or squamous cell carcinomas.

Further, in either adenocarcinomas or squamous cell carcinomas, the image analysis scores were higher in low PD-L1 cases, and lower in cases with high expression of PD-L1 (Fig. 4b–e). Noticeably, in both squamous cell carcinomas and adenocarcinomas, the differences between pathologists and image analysis were more obvious in cases with negative PD-L1(22C3) expression (Fig. 4d, e).

### Automated immune cells PD-L1 evaluation

**Fig. 5:**
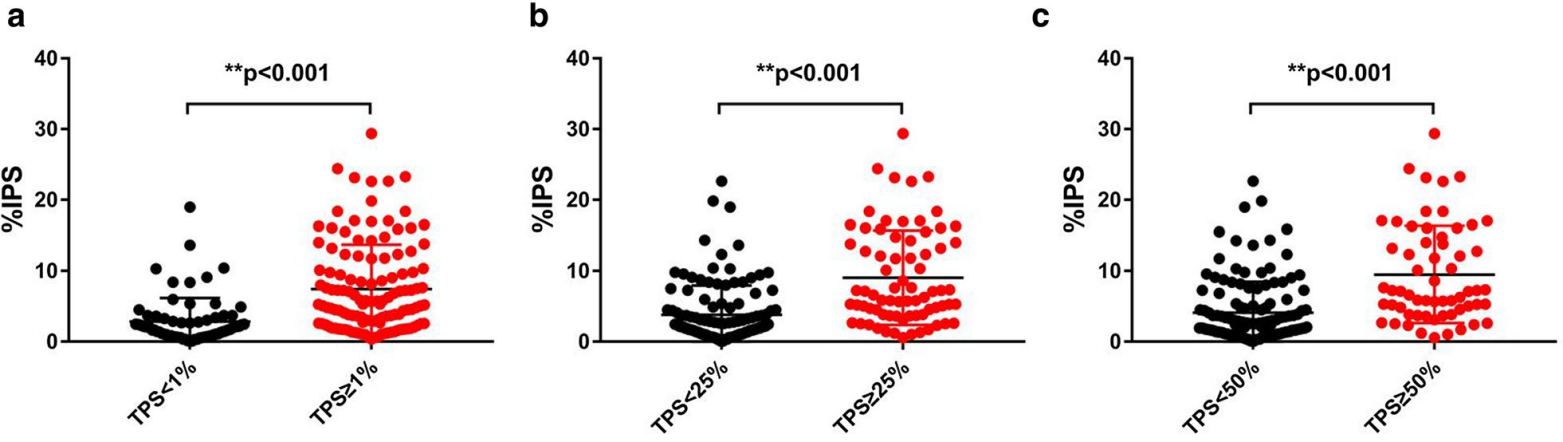
Correlation between TPS and IPS at 1% (**a**), 25% (**b**) and 50% (**c**) TPS cutoff values. *TPS* tumor proportional scoring, *IPS* immune cell proportion scoring

Besides tumor cells, we also optimized our algorithm for immune cells PD-L1(SP263) evaluation. In the 192 NSCLC PD-L1(SP263) staining slides, the automated IPS scores achieved high positive correlation with the pathologists TPS scores (Spearman = 0.531, Pearson = 0.494). At 1%, 25% and 50% TPS cutoff values, high IPS scores were significantly associated with high TPS cases (Fig. 5a–c). At the same time, we used 1% as TPS and IPS cutoff values simultaneously, and the result also demonstrated that high PD-L1 TPS scores were significantly associated with high IPS scores (*P* < 0.001, Additional file 1: Table S1).

### Comparison between C-Net and the previous established algorithms

Mi et al. reported the best performance so far for automated tumor proportion scoring [24]. Comparing the proposed C-Net against Mi et al. [24], U-Net [25] and S^3^Net [26], and verified the effectiveness of weighted pixel-wise cross-entropy loss on the Cell tags, we listed the obtained average performance of these models in Table 2. In close tumor cells, based on the construction of the proposed weighted pixel-wise cross-entropy loss, the C-Net was able to identify different tumor cells individually which helped achieve the best performance in the Object F1 Score, MAE, RMSE and MAPE [25] on the validation data of Cell tags. Meanwhile, due to the norm in the pro-posed loss strengthens to supervise the close tumor cells, the performance of the C-Net in terms of the object-level recall would be improved significantly. We visualized two patches images and the corresponding localization results obtained by different deep models, together with the ground truth, in Additional file 1: Figure S1.

**Table 2:** Comparison between our C-Net and the previous cellular localization algorithms on the cell data set

Method	F1 score			MAE			RMSE			MAPE		
	TC (+)	TC (−)	Avg.	TC (+)	TC (−)	Avg.	TC (+)	TC (−)	Avg.	TC (+)	TC (−)	Avg.
Mi	0.56	0.57	0.57	40.27	24.34	32.31	52.76	28.99	40.86	44.61	53.17	45.61
U-Net	0.58	0.64	0.61	39.71	20.01	29.86	48.93	26.01	37.37	43.82	42.85	43.34
S^3^ Net	0.68	0.71	0.70	30.07	17.52	23.80	42.68	23.53	33.11	33.82	38.16	35.99
C-Net	0.75	0.78	0.77	19.01	10.41	14.71	27.21	17.21	22.21	24.19	23.34	23.77

*F1 score* object F1 score, *MAE* mean absolute error, *MAPE* mean absolute percent error, *RMSE* root mean squared error

### Evaluation of R-Net effectiveness

To evaluate the effectiveness of R-Net algorithm, based on regional segmentation network, automated tumor proportion scoring was employed on big patches sampled from 61 WSIs, excluding the 30 WSIs in the training data set. The results demonstrated that combining R-Net could significantly improve the performance of all the cellular localization networks (Table 3 and Additional file 1: Figure S2). Specifically, the local cellular features of TC (+) and positive immune cells could hardly be used by a common CNN to classify cells correctly. Benefited from the R-Net contextual information was provided while classifying similar cells. Out results showed that R-Net could significantly improve the performance of each localization network. In addition, being trained by the same cell data set and combined with our R-Net, the concordance between our image analysis TPS values and pathologist scores was higher than with the other three methods (Table 3).

**Table 3:** Effectiveness of our R-Net and comparison between different cellular localization algorithms and pathologists on big patches

	R-Net	MAE	RMSE	PCCs	SRCC	ICC
Mi	–	12.34	17.82	0.834	0.921	0.917
	√	9.64	15.71	0.882	0.927	0.919
U-Net	–	10.22	15.45	0.865	0.936	0.935
	√	8.83	14.48	0.904	0.937	0.935
S^3^-Net	–	8.18	13.46	0.905	0.945	0.943
	√	7.56	12.73	0.928	0.956	0.953
C-Net	–	8.00	12.19	0.906	0.951	0.951
	√	7.55	11.82	0.933	0.965	0.963

*ICC* intraclass correlation coefficient, *MAE* mean absolute error, *MAPE* mean absolute percent error, *PCC* Pearson product-moment correlation coefficient, *RMSE* root mean squared error, *SRCC* Spearman’s rank correlation coefficient

## Discussion

In the current study, we showed that automated image analysis scoring algorithm can be used to determine tumor cell PD-L1 expression in patients with NSCLC and demonstrated high analytical concordance with pathologist scores. The image analysis algorithm revealed stronger yet comparable concordances at 10%, 25%, and 50% cutoffs, whereas the concordances were relatively weak at 1% and 5% cutoff values. Further observations revealed higher image analysis scores in PD-L1–negative expression cases. Additionally, the correlation of concordance values between pathologist scores and image analysis scores demonstrated variable results in different histological tissues. The concordances in squamous cell carcinomas were better than those in adenocarcinomas at high or moderate cutoff values, whereas the concordances in adenocarcinomas were better than those in squamous cell carcinomas at low cutoff values.

The IHC method and C-Net are the commonly preferred techniques by pathologists. However, these methods rarely distinguish the cellular features of samples such as TC (+) and positive normal cells (e.g., histocytes). Further, these methods have shortcomings such as dependence on fixation techniques and variability during interpretation of the results. The low sensitivity and low concordance rate of the assay might be due to higher incidence of false negative results (> 20%) based on IC or TC ≥ 25% and IC ≥ 25% threshold. Thus, when lack of sufficient staining, the incidence of false positive and false negative was commonly observed in locating and classifying tumor cells by C-Net alone. Hence, a novel automated TPS framework was proposed, which was based on a multistage ensemble strategy. We utilized the features of both C-Net and R-Net to design this multistage framework. C-Net predicted the cellular count ratio based TPS by quantitatively classifying, localizing, and counting the PD-L1 cell nuclei, whereas R-Net was used to generate a tumor probability map to distinguish tumor regions from their normal counterparts.

Our results were similar to a previously published study, which demonstrated that novel automated image analysis scoring algorithm was highly correlated with pathologist scores [21]. However, in our study the concordance between pathologists and image analysis algorithm was satisfied for 25% and 50% cutoff values, but the concordances of 1%, 5% and 10% cutoff values were lowered significantly. The reason might be that normal cells (such as histocytes) can be easily misdiagnosed with other variants of tumor cells. Image analysis distinguishes various tumor cells through PD-L1 immunohistochemical staining slides, which can lead to misdiagnosis of normal positive cells and higher image analysis scores in low-TPS-value cases, and misdiagnose of normal negative cells and lower scores in high-TPS-value cases. These results correlate with the study by Widmaier et al., where concordance was weak for lower cutoff pairs. This could be explained by the lower number of strongly positive cases and slightly lower specificity of low cutoff values evaluation [21]. Additionally, although we used PD-L1(SP263) staining slides for previous training, the concordances of PD-L1(22C3) staining slides were also satisfactory. Moreover, in the same series of sections, the concordances of PD-L1(SP263) slides were better. We noticed that the differences between pathologists and image analysis were more obvious in PD-L1(22C3) negative expression cases than in PD-L1(SP263) negative cases. It indicates that our image analysis can be potentially applied for different PD-L1 assays, although we still need optimization of the algorithm, especially in PD-L1 negative and low expression cases. Therefore, variable staining of slides and detailed annotations along with the development of image analysis algorithm were critical for improving the accuracy.

Our study showed that the concordances in squamous cell carcinomas cases were better than those in adenocarcinomas cases. This can be attributed to the fact that there are abundant histological variants of adenocarcinomas cases. Thus, more histological variants of adenocarcinoma samples are needed to test and improve the accuracy of the image analysis algorithm. Moreover, as noted earlier, the consistency in squamous cell carcinomas and adenocarcinomas varied at different cutoff evaluations. At low cutoff values, the concordances in adenocarcinomas were better. However, in case of moderate or high cutoff values, the concordances in squamous cell carcinomas were more satisfied than adenocarcinomas. This might be because of the differences in the rate of positive cases between squamous cell carcinomas and adenocarcinomas. Further observations are consistent with previously published studies [29, 30], where more PD-L1 high expression cases in squamous cell carcinomas were observed, which indicated that in the positive tumor cell or region training datasets, there were more squamous cell carcinoma tags than adenocarcinomas. At the same time, more negative adenocarcinoma tags were included in the training dataset. Moreover, previously published studies exhibited that PD-L1 expression was significantly higher in the more aggressive variants of adenocarcinomas (e.g., papillary and solid types) than in the common others (e.g., lepidic and acinar types) [29–31]. It should be noted that the ratio of PD-L1 staining was different in various variants of adenocarcinomas.

Besides tumor cells, PD-L1 expression of immune cells also influence the effectiveness of immunotherapy [1]. However, compared with TPS, the concordances of IPS by different pathologists were low [13]. We optimized our image analysis for IPS evaluation. The result demonstrated that high IPS scores were significantly associated with high TPS cases. As same as our results, the previous researches have mentioned that high PD-L1 expression in immune cells was significantly associated with high PD-L1 level in tumor [32, 33]. Actually, there were limited research about automated IPS evaluation. This is partially due to the difficulties of recognizing and distinguishing tumor regions and tumor related immune cells. Our research revealed that combining R-Net and C-Net could improve the accuracy of automated IPS evaluation. However, we need assess the concordances between pathologists and image analysis IPS scores directly in the future.

There are several limitations in this study. First, the training or validation samples used for our research were core biopsy or large section samples. Cytology samples of patients in advanced stage of disease like fine needle aspiration biopsy, bronchoalveolar lavage fluid, and hydrothorax samples are required to further understand the efficiency of the image analysis [34]. Unfortunately, none of the pivotal clinical trials included cytology specimens for the development of the companion PD-L1 IHC assays [13]. Nevertheless, a series of studies have reported on the concordance of PD-L1 assessment on cytology versus matching surgical specimens, and tumor cell PD-L1 scoring between different PD-L1 IHC assays in cytology samples were reliable based on literature report [35–37]. Thus, utilization of our scoring algorithm in cytology samples are promising though further training and validation are warranted. In addition, earlier studies demonstrated that the PD-L1 expression between primary and metastatic tumors was discordant [38], and the PD-L1 expression of metastatic tumors was also associated with either immunotherapy response or survival [39]. Furthermore, as our training and validating cases consisted of primary tumors, recognizing positive and negative tumor cells among whole sample images would be a challenge.

## Conclusion

In conclusion, the proposed automated TPS system based on image analysis algorithm comprising C-Net with a weighted pixel-wise cross-entropy as loss function and R-Net achieved a comparable concordance with pathologist scores. As observed in a previously published study [21], the concordances at high cutoff values were better than at low cutoff values. The concordances in squamous cell carcinomas and adenocarcinomas varied at different cutoff evaluations. In addition, our research revealed that combining R-Net and C-Net could potentially improve the accuracy of automated IPS evaluation.

## Supplementary Information

**Additional file 1: Figure S1.** Visualization of tumor cell localization results of 2 different histological variant cases selected from the cell data sets. TC (+) (red) and TC (−) (green) are highlighted by different colors. The yellow circle area illustrates that C-Net improves the specificity of tumor cells recognition. Blue circle represents the false recognition of normal cells as tumor cells. **Figure S2.** Visualization of tumor cell localization results of a case selected from the cell data sets. (A) Original image and (B) Pathologist annotation; (C) Comparison between cell localization results with and without R-Net illustrated that combining with R-Net could improve the accuracy of cell localization algorithms. TC (+) (red), TC (−) (green), histocytes (blue), and stromal cells (yellow) were highlighted by different colors. The blue curve represented the histocytes region. **Table S1.** Correlation between TPS and IPS.

## Data Availability

The datasets during and/or analysed during the current study available from the corresponding author on reasonable request.
