# Supplementary material for: Automated tumor proportion scoring for PD-L1 expression based on multistage ensemble strategy in non-small cell lung cancer
Source: J Transl Med. 2021 Jun 7;19:249. doi: 10.1186/s12967-021-02898-z (PMC8185941; doi:10.1186/s12967-021-02898-z)
Supplement: Supplementary file 1 — Additional file 1: Figure S1. Visualization of tumor cell localization results of 2 different histological variant cases selected from the cell data sets. TC (+) (red) and TC (−) (green) are highlighted by different colors. The yellow circle area illustrates that C-Net improves the specificity of tumor cells recognition. Blue circle represents the false recognition of normal cells as tumor cells. Figure S2. Visualization of tumor cell localization results of a case selected from the cell data sets. (A) Original image and (B) Pathologist annotation; (C) Comparison between cell localization results with and without R-Net illustrated that combining with R-Net could improve the accuracy of cell localization algorithms. TC (+) (red), TC (−) (green), histocytes (blue), and stromal cells (yellow) were highlighted by different colors. The blue curve represented the histocytes region. Table S1. Correlation between TPS and IPS. [file 12967_2021_2898_MOESM1_ESM.docx]

**Additional file 1**

**Figure S1.** Visualization of tumor cell localization results of 2 different histological variant cases selected from the cell data sets. TC (+) (red) and TC (-) (green) are highlighted by different colors. The yellow circle area illustrates that C-Net improves the specificity of tumor cells recognition. Blue circle represents the false recognition of normal cells as tumor cells.


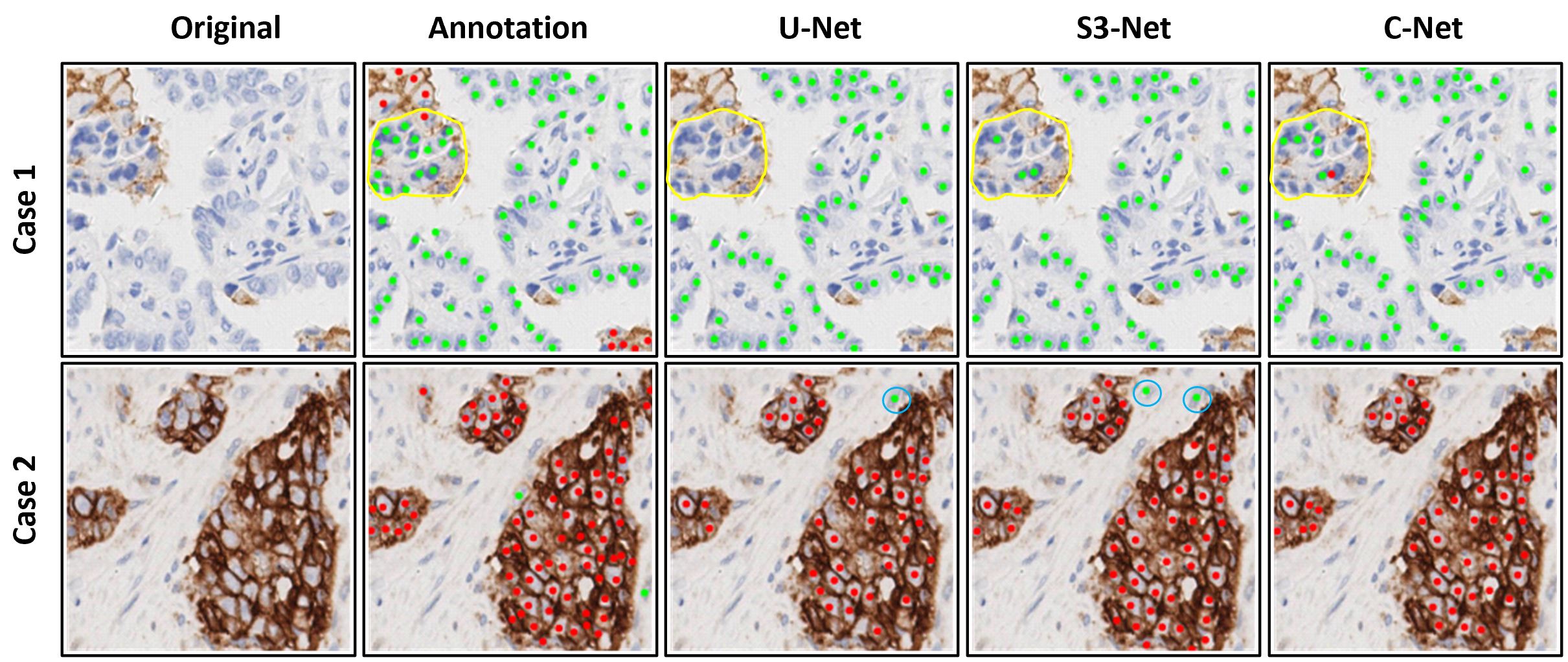


**Figure S2.** Visualization of tumor cell localization results of a case selected from the cell data sets. (A) Original image and (B) Pathologist annotation; (C) Comparison between cell localization results with and without R-Net illustrated that combining with R-Net could improve the accuracy of cell localization algorithms.

TC(+) (red), TC(−) (green), histocytes (blue), and stromal cells (yellow) were highlighted by different colors. The blue curve represented the histocytes region.


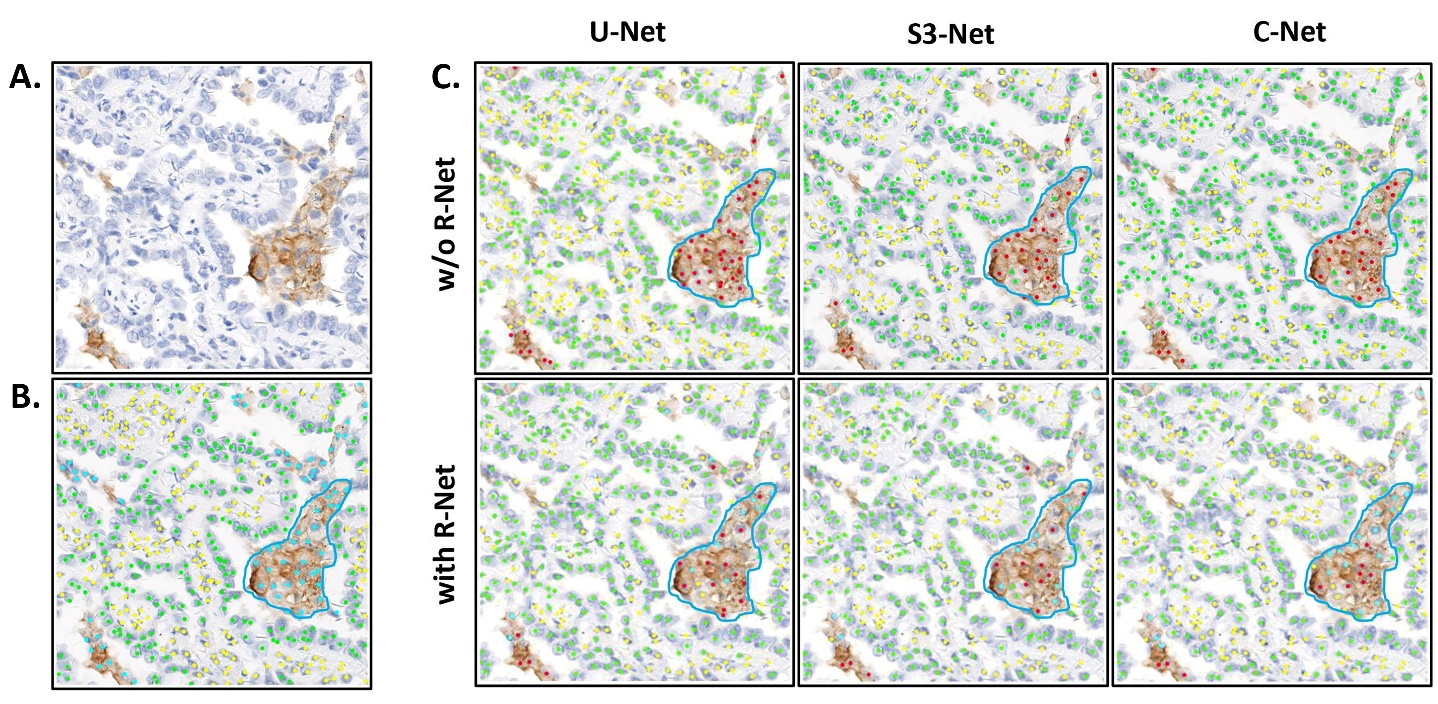


**Table S1.** Correlation between TPS and IPS.

|  | **Total (n=192)** | **IPS high (≥1%)** | **IPS low (<1%)** | **p** |
| --- | --- | --- | --- | --- |
| **TPS high (≥1%)** | 122 | 118 | 4 | <0.001 |
| **TPS low (<1%)** | 70 | 50 | 20 |  |

Abbreviations: TPS, tumor proportional scoring; IPS, immune cell proportion scoring
